# Application of smart solid lipid nanoparticles to enhance the efficacy of 5-fluorouracil in the treatment of colorectal cancer

**DOI:** 10.1038/s41598-020-73218-6

**Published:** 2020-10-12

**Authors:** Taylor Smith, Kevin Affram, Ebony L. Nottingham, Bo Han, Felix Amissah, Sunil Krishnan, Jose Trevino, Edward Agyare

**Affiliations:** 1 College of Pharmacy and Pharmaceutical Sciences, Florida A&M University, 1415 South Martin Luther King Blvd, Tallahassee, FL 32307 USA; 2 Department of Surgery, Keck School of Medicine University of Southern California, Los Angeles, CA USA; 3 College of Pharmacy, Ferris State University, Big Rapids, MI USA; 4 Mayo Clinic, Jacksonville, FL USA; 5 Department of Surgery, College of Medicine, University of Florida, Gainesville, FL USA

**Keywords:** Cancer, Nanoscience and technology

## Abstract

5-Fluorouracil (5-FU) is a standard treatment option for colorectal cancer (CRC) but its rapid metabolism and systemic instability (short half-life) has hindered its therapeutic efficacy. The objective of this study was to develop a novel drug delivery system, solid lipid nanoparticle (SLN), capable of delivering high payload of 5-FU to treat CRC. The rational was to improve 5FU-nanocarrier compatibility and therapeutic efficacy. The SLN-loaded 5-FU was developed by utilizing a Strategic and unique Method to Advance and Refine the Treatment (SMART) of CRC through hot and cold homogenization approach. The SLN was made of unique PEGylated lipids and combination of the surfactants. Cytotoxicity studies, clonogenic assay, flow cytometry and confocal imaging were conducted to evaluate the effectiveness and cellular uptake of 5FU-SLN_4_ in HCT-116 cancer cells. Pharmacokinetic (PK) parameters and receptor expressions were determined while tumor efficacy studies were conducted on mouse bearing subcutaneous HCT-116 cancer. Among the all the formulations, 5FU-SLN_4_ was the most effective with particle size of was 263 ± 3 nm, zeta potential was 0.1 ± 0.02 and entrapment efficiency of 81 ± 10%. The IC_50_ value of 5FU-SLN_4_ (7.4 ± 0.02 µM) was 2.3 fold low compared with 5-FU (17.7 ± 0.03 µM). For tumor efficacy studies, 5FU-SLN_4_ significantly inhibited tumor growth in comparison to 5-FU while area-under plasma concentration-time curve (AUC) of 5FU-SLN_4_ was 3.6 fold high compared with 5-FU. HER2 receptors expression were markedly reduced in 5-FU-SLN_4_ treated mice compared with 5FU and liver and kidney tissues showed no toxicity at dose of 20 mg/kg. 5FU-SLN_4_ was highly cytotoxic against HCT-116 cells and significantly inhibited subcutaneous tumor growth in mice compared with 5-FU. This emphasizes the significance of developing a smart nano-delivery system to optimize the delivery efficiency of anticancer drugs to tumors.

## Introduction

CRC is the third most diagnosed cancer in individuals in US, and a third leading cause of cancer related deaths. CRC is the second highest cause of cancer deaths when men and women are combined^1^. According to many sources, CRC is expected to cause more than 50,000 deaths during 2019^1,2^.

The present treatment of CRC largely depends on radiotherapy, surgery, targeted therapy and chemotherapy; however, surgery is the preferred option if CRC is at stages one through to four with or without chemotherapy and/or radiotherapy^3^. Based on the stage of CRC, two or more treatment options are combined to achieve the desired therapeutic effect. Further, reports show that 5-year survival rate of individuals with metastatic cancer is less than those without metastatic cancer^1^. The present treatment strategies, examples, targeted therapy and chemotherapy largely affect CRC cells mainly through cell senescence and apoptosis.

The existing therapeutic agents for the treatment of CRC are diverse. They include cytotoxic agents such as 5-FU, oxaliplatin, capecitabine and irinotecan, and targeted therapies such as bevacizumab and anti-epidermal growth factor receptor anti-bodies (examples are cetuximab and panitumumab).

While 5-FU is often used alone or in combination with other anticancer agents such as irinotecan and folinic acid (FOLFIRI) or folinic acid and oxaliplatin (FOLFOX)^4^, it’s antimetabolite mimics uracil and thymine which eventually incorporates into DNA and RNA synthesis leading to cytotoxicity and cell death^5^. 5-FU is a fluorinated pyrimidine that inhibits DNA and RNA activity through inhibition of the rate-limiting enzyme (thymidylate synthetase) in pyrimidine nucleotide synthesis^6^.

Although 5-FU shows inhibitory activity on DNA synthesis, its therapeutic efficacy is limited due to its hydrophilic nature and rapid metabolism by dihydropyrimidine dehydrogenase. Therefore, continuous administration of high doses of 5-FU must be applied to attain the required therapeutic concentration which causes severe toxic effects on the gastrointestinal tract, hematological, neural, cardiac and dermatological reactions^7^. Hence, there is a need to develop a smart delivery system capable of protecting and delivering a high payload of 5-FU to the desired site with improved therapeutic efficacy.

Nanotechnology has become one of the fastest growing areas with potential applications in drug therapy such as liposomes, micelles, polymeric nanoparticles, and dendrimers have been widely used to convey and overcome drugs with aqueous solubility or systemic stability related issues. Among all the nanodelivery systems, lipid-based nanocarriers are commonly considered the least toxic for in vivo applications and a remarkable progress has been achieved in DNA/RNA in delivery^8^.

While delivery approaches such as liposomal and polymeric nanoparticles have had setbacks, investigations of solid lipid nanoparticle (SLN) as substitute drug carrier system has proven to be a novel, due to its various advantages such as the ability to incorporate of both hydrophilic and lipophilic drugs, ease of scale-up and enhanced physical stability^9,10^. By virtue of nanoparticle’s size effect and improved physicochemical properties, the SLN can extravasate into the tumor through the tumor’s leaky vasculature and increase localized tumor drug exposure^11^.

The current study aimed to develop a smart delivery system capable of delivery a high payload of 5-FU, a hydrophilic drug, into optimized SLNs (5FU-SLNs) with special emphasis on protection and prolonging systemic circulation of 5-FU and, to improve its therapeutic outcome. In this context, the use of smart PEGylated 5FU-SLN_4_ was developed and optimized to obtain a high entrapment efficiency that was highly cytotoxic against HCT-116 cells, improved 5-FU pharmacokinetic parameters and significantly inhibited subcutaneous tumor growth in mice compared with 5-FU. These improved efficacy of 5-FU emphasized the significance of developing a smart nano-delivery system to optimize the delivery efficiency of anticancer drugs to tumors.

## Results

CRC mortality has slowly decline largely due to cancer prevention and early detection, however; cancer prevention and early detection may not be the only key factors for the management or treatment of patients with CRC. In 2019, 145,600 estimated new cases of CRC will occur and an estimated 51,020 deaths is expected to occur for the year^12^. While prevention through screening is the best approach to combat the development of CRC, many patients present with advanced disease that will require surgery and systemic therapy to improve survival. With respect to systemic therapy, the median survival of patients with metastatic CRC has improved over the past three decades due to the introduction of 5-FU.

5-FU is commonly used alone or in combination with other anticancer drugs as first—line treatment for patients who have developed advanced CRC. The anticancer agent exerts its effect by interrupting RNA processing and blocking normal DNA synthesis. However, the response rate of 5-FU is less than 15% and due to dihydropyrimindine dehydrogenase (DPD) 80% of 5-FU is rapidly broken down limiting the therapeutic efficacy of the anti-cancer agent^13–15^.

Pharmaceutical nano drug systems have become idea for the transport of anti-cancer drugs and the reduction of unwanted distribution and side effects to healthy cells. These nanoparticles provide protection for anti-cancer drugs from first—pass metabolism and enzymatic degradation. In addition, nano drug systems generally provide enhancement properties of the drug, for example, prolong systemic circulation, increase drug entrapment and loading capacity. For many of those reasons, we decided to develop a novel SLN delivery system capable of high payload of 5-FU and improve 5-FU therapeutic efficacy on HCT-116 colon cancer.

### Characterization of PEGylated 5FU-SLNs

**Figure 1:**
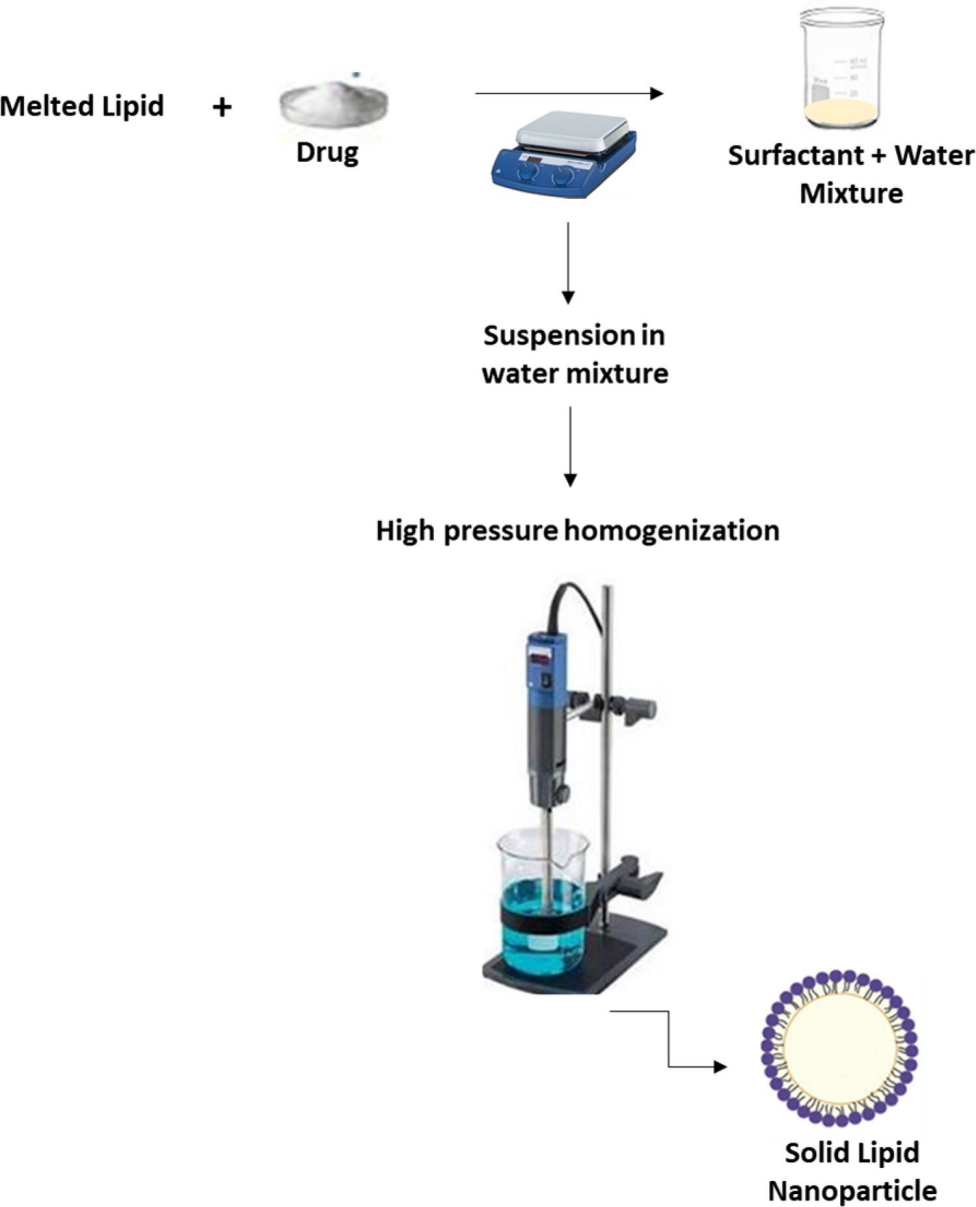
Nanoparticle schematic: a visual illustration demonstrating the sequence of 5-FU loaded solid lipid nanoparticles.

**Figure 2:**
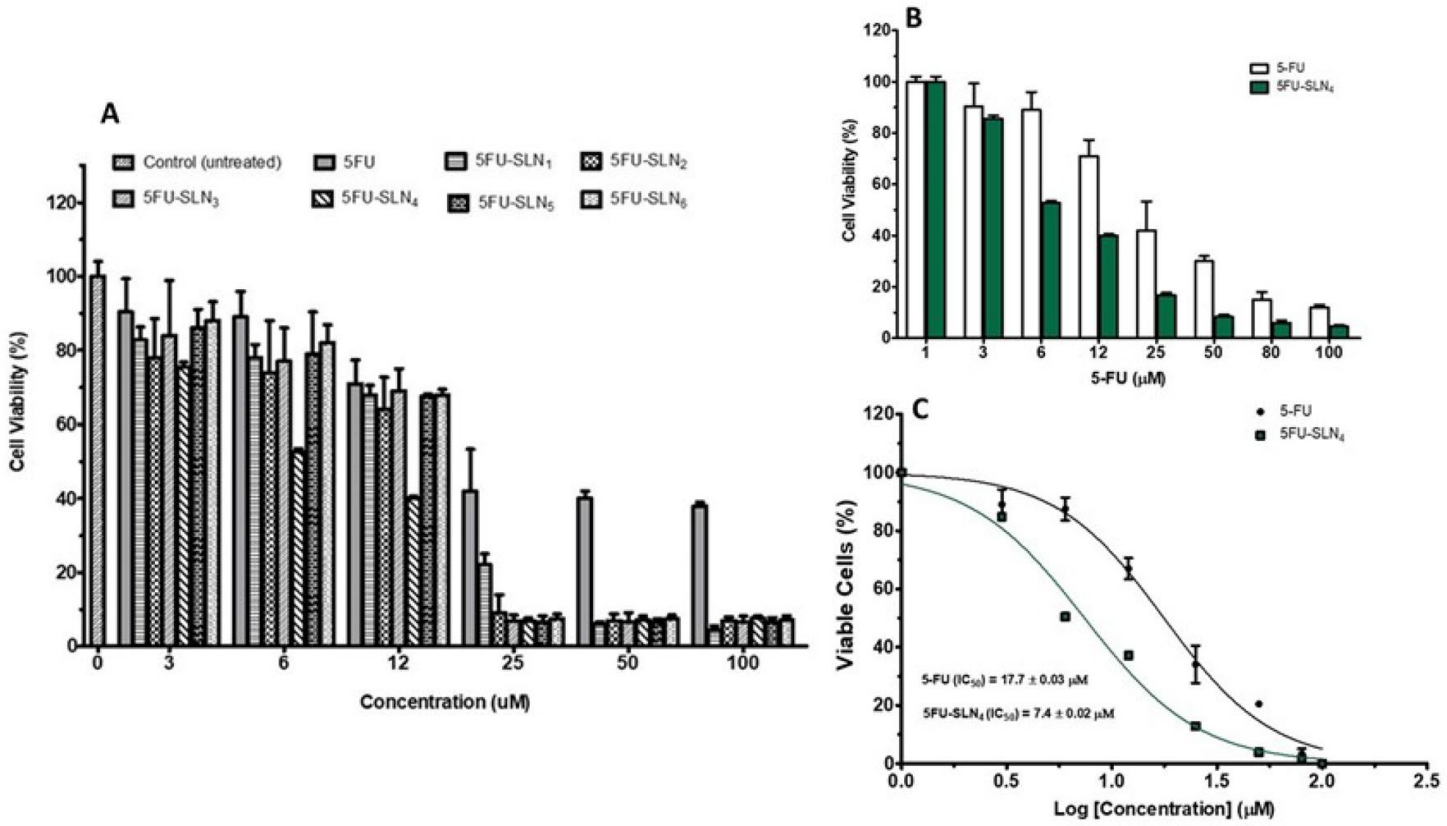
Cell viability: Effects of 5FU-SLN formulations on HCT-116 cell growth. (**A**) Percent viability of HCT-116 cells after treatment with 5-FU and different 5FU-SLN formulations. (**B**) Viability of HCT-116 cells after treatment with 5-FU and 5FU-SLN_4_, treatment shows dose-dependent growth inhibition of cell lines, and (**C**) IC_50_ values generated based on non-linear curve fitting for dose–response curves for 5-FU and 5FU-SLN_4_ treated HCT-116 cells.

PEGylated SLNs are carrier systems that have been developed to encapsulate, protect and deliver active drug components. Six different batches of loaded SLNs were prepared by cold homogenization method. Cold homogenization was used to overcome temperature induced drug degradation, drug distribution into aqueous phase and crystallization modification as the method is also the preferred technique for hydrophilic drugs^16,17^. The active drug molecules were dispersed into the lipid melt fabricating a drug enriched core, a type of SLN as illustrated in Fig. 1. All batches were used in initial in-vitro studies indicated in Fig. 2A. Table 1 presents the drug, lipid, and surfactant composition for each batch of 5FU-SLN**.** With the chosen lipid compositions, glycerol monostearate, Compritol 888 ATO and Precirol ATO 5 (2–3%) the particle size and dispersity stayed in range, 77–288 nm. 5FU-SLN_1_ had a glycerol monostearate content of 2% and an observed higher particle size with decreased dispersity compared to other formulations with 3% lipid content. The choice of surfactants and its concentration has a great impact on SLN size. Lipid and surfactant compositions were kept below 5% to prevent particle growth and maintain small particle size. Again, higher concentrations of surfactants may lead to toxicity as reported in literature that ionic emulsifiers are more likely to produce smaller size in comparison to non-ionic emulsifiers even at the equal concentrations^18^. With the knowledge given formulations 5FU-SLN_5_ and 5FU-SLN_6_ both contain a non-ionic emulsifier as well as Lecithin, a highly recommended co-emulsifier to help decrease the particle size. The results showed smaller particle size for those formulations in comparison to those that were prepared with frequently used Tween 80 only. The surfactant concentrations did not drastically change/effect zeta potential. The results suggest that the surfactant concentrations are sufficient for the development of SLNs as the particle size range is expected. It could be seen from Table 1 the mean particle size, zeta potential and polydispersity index (PI) for each formulation (Supplementary Figures S7 and S8). The PI is used to measure the degree of non-uniformity of a size distribution of nanoparticles. With the use of the cold homogenization technique, wide dispersion typically occurs as indicated by the PI values shown in Table 1. Although all formulations had reasonable particle sizes, ranging from 77 to 288 nm, 5FU-SLN_4_ with particle size of 263 ± 3 nm with transmission electronic microscopy image (TEM) as shown in Supplementary Figure S1 was chosen for this study. The reason for selecting 5FU-SLN_4_ was due to the desired cytotoxicity activity displayed by 5FU-SLN_4_ in Fig. 2A–C, Supplementary figure S6 as well the high entrapment efficiency (EE) of 81 ± 10% (Table 1 and Supplementary Table S1).

**Table 1:** Composition and characterization of 5FU-SLN.

Formulation	5-FU (%w/v)	Lipid composition	Lipid ratio (%w/v)	Surfactant composition	Surfactant ratio (%w/v)	Mean particle size (nm)	Mean zeta potential (mV)	Polydispersity index (P.I)
5FU-SLN_1_	0.15	GM	2	TWN 80	1.5	288 ± 5	0.1 ± 0.02	0.3
5FU-SLN_2_	0.15	Compritol	3	TWN 80	4	170 ± 3	0.2 ± 0.05	1.4
5FU-SLN_3_	0.15	Precirol	3	TWN 80	4	228 ± 6	0.6 ± 0.02	1.2
5FU-SLN_4_	0.15	Precirol	3	TWN80/Lecithin	4:2	263 ± 3	1.1 ± 0.02	0.9
5FU-SLN_5_	0.15	Precirol	3	Poly/Lecithin	4:2	130 ± 5	0.5 ± 0.03	0.7
5FU-SLN_6_	0.15	Compritol	3	Poly/Lecithin	4:2	77 ± 3	0.2 ± 0.05	2.8

All the formulations were freshly prepared.

TWN 80, Tween 80; GM, Glycerol monostearate; Poly, Poloxamer.

### In vitro cell viability studies

To evaluate the efficacy of PEGylated 5FU-SLNs, in vitro cytotoxicity of 5-FU and various PEGylated 5FU-SLN formulations studies were performed against HCT-116 cells for 48 h at different concentrations. Figure 2A shows the effect of increasing concentration of 5-FU along with the six batches (5FU-SLN_1_, 5FU-SLN_2,_ 5FU-SLN_3,_ 5FU-SLN_4._5FU-SLN_5_ and 5FU-SLN_6_) to assess the growth inhibition of HCT-116 cancer cells. As shown in Fig. 2, PEGylated 5FU-SLN_4_ was most effective against HCT-116 cell growth at all concentrations in comparison to the free 5-FU (Fig. 2B) and the five 5FU-SLN formulations (Fig. 2A). To determine the extent of HCT-116 cells sensitivity to 5-FU and 5FU-SLN_4_, half maximum inhibitory concentrations (IC_50_) of 5-FU and PEGylated 5FU-SLN_4_ against HCT-116 cells were measured. Figure 2C shows that IC_50_ value (17.7 ± 0.03 μM) of 5-FU was 2.4 fold-high compared with that of 5FU-SLN_4_ (7.4 ± 0.02 μM) suggesting that 5FU-SLN_4_ was significantly effective and would require significantly less concentration to treat HCT-116 cells compared with 5-FU.

### In vitro drug release kinetics from PEGylated SLNs

**Figure 3:**
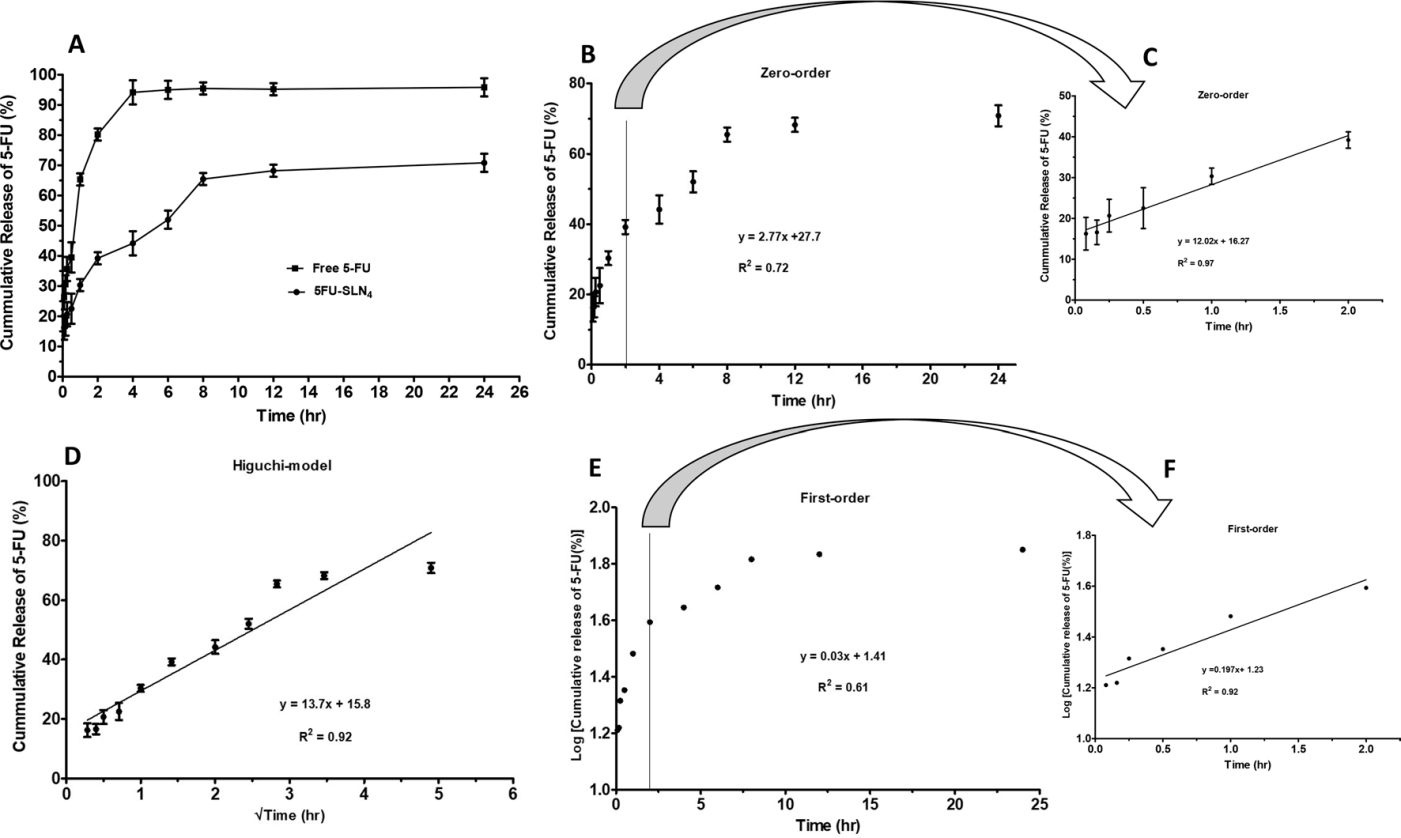
In-vitro release kinetics of 5FU-SLN_4_: (**A**) In-vitro cumulative release profile of 5-FU from SLN_4_ at for 24 h, in which free 5-FU was the control. (**B**) The release profile of 5-FU exhibiting zero-order kinetics, (**C**) The release kinetics for the first 2 h (extracted from **B**), the release followed a linear pattern with R^2^ = 0.97, (**D**) The release profile of 5-FU exhibiting Higuchi-model release. The 5-FU release from SLN_4_ indicates Higuchi-model release with a R^2^ = 0.92, (**E**) The release profile of 5-FU exhibiting first-order kinetics, (**F**) Release kinetics for the first 2 h (extracted from **E**), the release followed a linear pattern with R^2^ = 0.92.

In Fig. 3, the cumulative in-vitro drug release of 5-FU during a 24 h period of incubation at 37 °C was presented. In this case, the dialysis bag method was used to study in vitro evaluation of 5-FU release profile from 5FU-SLN_4_. The formulation was designed with the expectation that 5-FU was encapsulated and retained within the SLN_4_. The release of free 5-FU was rapid and almost complete from the dialysis bag with about 80% released just within 2 h and about 90% released within the first 3 h. The free 5-FU release continued until it reached 95% within the first 4 h. This suggests that any free 5-FU placed in the dialysis bag would rapidly diffuse out in a significant amount within a short period of time. In addition, its release profile could be used to differentiate the release pattern of 5-FU from SLN_4_ as long as sink condition was maintained and diffusion of 5-FU to the receiving medium depended on concentration gradient (Fig. 3A). In comparison to 5-FU release from SLN_4,_ about 40% of 5-FU was released within the first 2 h followed by a gradual or slow release of additional 20% over a period of 6 h and reached steady state at time 8 h. This implies that most of the 5-FU remained entrapped in SLN_4_ under the study conditions (Fig. 3A).

To determine the 5-FU diffusion mechanism, in vitro release kinetics of 5-FU from SLN_4_ was modeled as shown in Fig. 3B,C for zero-order kinetics, Fig. 3D for Higuchi model and Fig. 2E,F for first-order kinetics. The best-fit release mechanism was determined based on the comparison of R^2^ values of the various kinetic models (Fig. 3B,D,E)^19^. The R^2^ value (0.92) of Higuchi model (Fig. 3D) was found to be the highest compared with R^2^ value (0.72) of zero-order (Fig. 3B) and R^2^ value (0.61) of first-order (Fig. 3E) based on the 24 h. This suggests that 5-FU released from SLN_4_ followed Higuchi diffusion kinetics and implies that 5-FU release come from a homogeneous delivery system and diffuses out of the delivery system over a period of time^19,20^. Figure 3C (R^2^ = 0.97) and Fig. 3F (R^2^ = 0.97) were not considered in determining the best-fit model of 5-FU release because the graphs covered only the first 2 h (first six data points).

### Flow cytometry and confocal imaging

To further assess PEGylated 5FU-SLN_4_ cell uptake by HCT-116 cells, flow cytometry analysis was performed. Supplementary Figure S2 shows cellular uptake of SLN as they were tagged with rhodamine (Rho-SLN_4_) and incubated for 3 h at 37 °C. As shown in Supplementary Figure S2 (A and B), flow cytometry data revealed significant Rho-SLN cellular uptake**.** To further confirm the uptake of PEGylated 5FU-SLN_4_, HCT-116 cells were treated for 24 h at 37 °C with FITC-labeled SLNs. Confocal images Supplementary Figure S2C of HCT-116 cells showed uptake of FITC-SLN as Hoechst dye was used to counterstain nuclei. The merged image indicated that the solid lipid nanoparticle localized within the cell nuclei.

### Clonogenic studies

Clonogenic survival assay was conducted to evaluate in vitro cell survival. The primary goal was to determine the proliferative ability of HCT-116 cell lines after treatment with 5-FU and 5FU-SLN_4_. As shown in Supplementary Figure S3A, as concentrations of both 5-FU and 5FU-SLN_4_ increases, survival percent decreases. More importantly as 5FU-SLN_4_ concentration increased from 3 to 25 μM, we observed a significantly decreased colony formation of 5FU-SLN_4_ treated HCT-116 cells as shown in Supplementary Figure S3B. In contrast, colony formation especially in 6 μM 5-FU treated HCT-116 cells was a remarkably higher than that treated with 5FU-SLN_4._ The findings seem to suggest that a significant DNA damage might have occurred in 5FU-SLN_4_ treated cells than that 5-FU and was most likely due to the HCT-116 cells inability to repair DNA and reproduce.

### Western blot analysis

**Figure 4:**
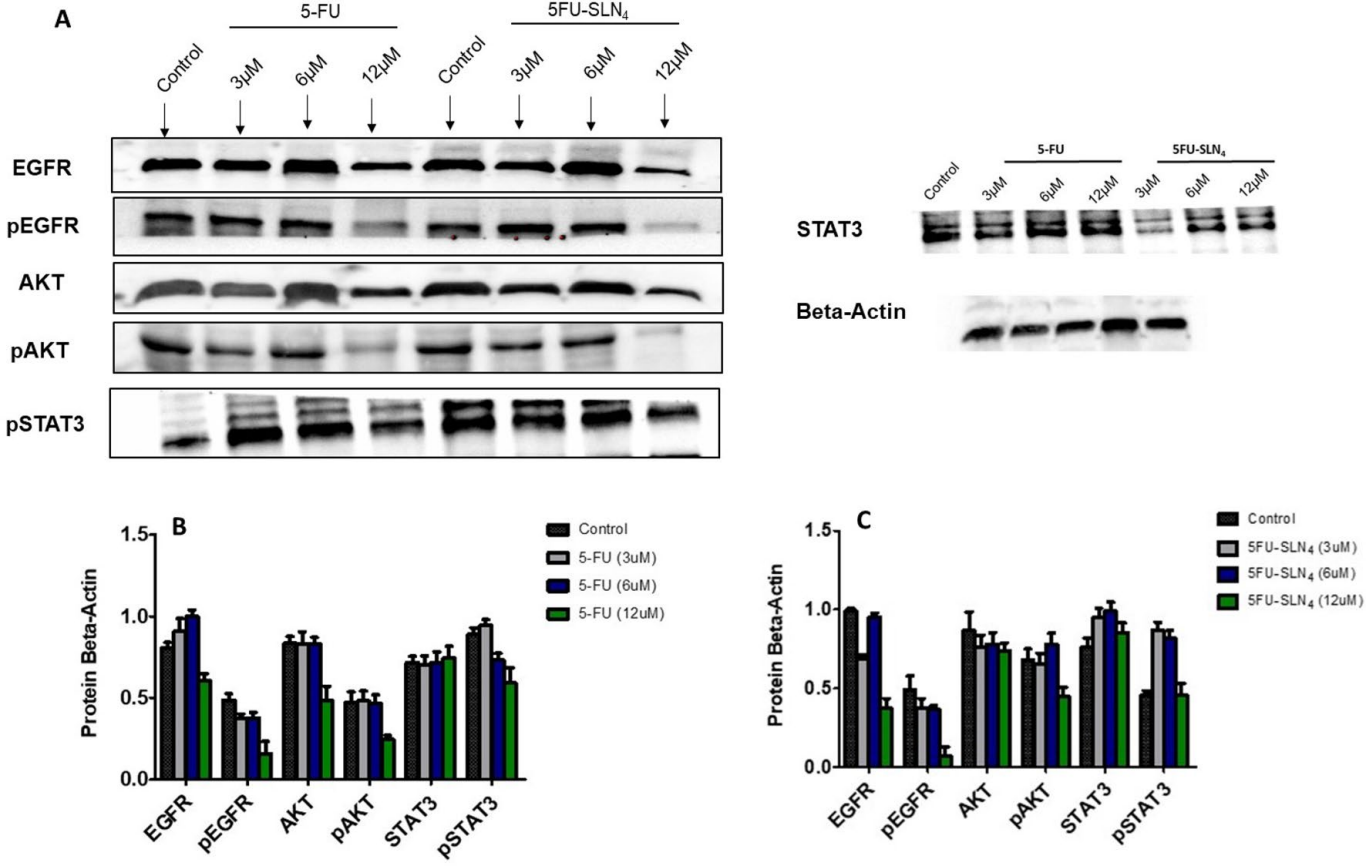
Cellular apoptosis by 5FU-SLN_4_: Western blot analysis shows the protein expression affected after 48 h of treatment with 12 µM of 5FU-SLN_4_ (**A**). Beta-Actin was used as a loading control. Effects of 5-FU on protein beta-actin ratio and protein expression in HCT-116 cells (**B**). Effects of 5FU-SLN_4_ on protein beta-actin ratio and protein expression in HCT-116 cells (**C**). Membrane blots were cut to enable blotting for multiple antibodies. Data presented as mean ± SD.

Utilizing the inhibitory concentrations (IC_50_) from viability experiments, we performed protein expression analysis in HCT-116 cells treated with 5-FU and PEGylated 5FU-SLN_4_ for 48 h. In Fig. 4A protein analysis showed greater dose-dependent reduction of EGFR and AKT phosphorylation with treatment of PEGylated 5FU-SLN_4_. As studies have shown, EGFR is highly expressed in advanced CRC with associated poor survival^21^. More importantly, the results indicate that, our developed formulation, PEGylated 5FU-SLN_4_ appeared to be more effective in targeting EGFR as seen in the reduction of protein expression compared with that of 5-FU treated cells (Fig. 4B,C). Our novel formulation, PEGylated 5FU-SLN_4_ reduced the expression of both the total and phosphorylated EGFR as well as some of the crucial molecules in the EGFR signaling pathways, activated AKT and STAT3. Such inhibitory effects of 5FU-SLN_4_ may negatively impacts the signaling through the key transduction pathways activated by EGFR such as RAS, phosphatidylinositol 3-kinase (PI3K), Src and signal transducer and activator of transcription 3 (STAT3) involved in the control of cell proliferation, survival and differentiation^22^. Blocking the EGFR-induced activation of the RAS-Raf-MEK-MAP kinase cascade or PI3K-AKT results in the inhibition of transcription factors regulating gene expression, while inactivation of STAT3 prevents dimerization and translocation to the nucleus where they act as transcription factors^23^.

### Acute toxicity

Clinical usefulness of 5-FU has been precluded because of its hepatotoxic and nephrotoxic side effects^24^. In the present study, careful examination of the H&E-stained sections of the liver and kidney of mice treated with 5-FU and 5FU-SLN_4_ (dose: 20 mg/kg) revealed normal histological (hepatic and renal) architecture compared with that of the control mice (Supplementary Figure S4). While other reports indicate loss of normal hepatic and renal architecture of mouse treated with 5-FU (80 mg/kg, dose)^25,26^, we did not observe any abnormality in hepatocytes and nephrocytes architecture of mice treated with either 5-FU or 5FU-SLN_4_ (dose: 20 mg/kg). More importantly, at a dose of 20 mg/kg 5FU-SLN_4_ significant CRC tumor growth suppression was observed, suggesting that the dose was tolerable and more effective than the 5-FU in the mice.

### Pharmacokinetics

5-FU and 5FU-SLN_4_ was intraperitoneally dosed (20 mg/kg) with a single bolus administration in mice to evaluate and compare the pharmacokinetic parameters of 5-FU and 5FU-SLN_4_ (at 5-FU equivalent dose). As shown in Table 2, the half-life (t_1/2_) of 5FU-SLN_4_ (2.13 ± 0.04 μg/mL * h) was found to be threefold high compared with 5-FU (0.68 ± 0.02 μg/mL * h) with *p*-value of 0.0001, while area under curve (AUC) of 5FU-SLN_4_ (54.7 ± 3.2 (μg/mL * h)) was significantly higher than 5-FU (15.5 ± 1.9 μg/mL * h) with *p*-value of 0.0001. As expected, the plasma clearance () of 5-FU (12.5 ± 1.3 mL/h) was remarkably high compared with 5FU-SLN_4_ (4.1 ± 0.8 mL/h) with *p*-value of 0.0007. In comparison with literature on pharmacokinetic profiles of similar 5-FU loaded SLNs formulations, we found that AUC and t_1/2_ values of our formulation 5FU-SLN_4_ were significantly greater than in literature^27–29^. In addition, CL value of 5FU-SLN_4_ was notably lower than that found in literature^27,29^. Put together, the results suggest that SLN_4_ may have prolonged plasma circulation of 5-FU and the observed favorable pharmacokinetics of 5-FU was mostly due to the unique ability of SLN_4_ to protect and deliver high payload of 5-FU. However, more studies will have to be conducted to elucidate the mechanisms underlying the 5FU-SLN_4_’s ability to increase the systemic exposure of 5-FU when administered intraperitoneally.

**Table 2:** Pharmacokinetic parameters of free 5-FU and 5FU-SLN_4_ upon IP injection in the mice.

Parameter	Unit	One-compartment model		Significance level *p*-value
		5-FU	5FU-SLN_4_	
k_a_	1/h	1.36 ± 0.02	1.91 ± 0.04	0.0001
K_10_	1/h	1.01 ± 0.03	0.32 ± 0.02	0.0001
t_1/2_	h	0.68 ± 0.02	2.13 ± 0.04	0.0001
V_d_	mL	14.3 ± 2.8	12.6 ± 0.6	0.3619
CL	mL/h	12.5 ± 1.3	4.1 ± 0.8	0.0007
C_max_	µg/mL	6.7 ± 0.4	12.7 ± 0.9	0.0005
T_max_	h	0.84 ± 0.06	1.12 ± 0.04	0.0025
AUC_(0 − t)_	µg/mL * h	15.5 ± 1.9	54.7 ± 3.2	0.0001
MRT	h	1.7 ± 0.4	3.6 ± 0.8	0.02

C_max_, peak plasma concentration; T_max_, peak time; AUC_(0 − t)_, area under the plasma concentration–time curve; MRT, mean residence time; k_10_, elimination rate constant; Cl, clearance; V_d_, volume of distribution; k_a_, absorption rate constant (Data analyzed using unpaired t-test, two-tailed).

### Tumor efficacy studies

**Figure 5:**
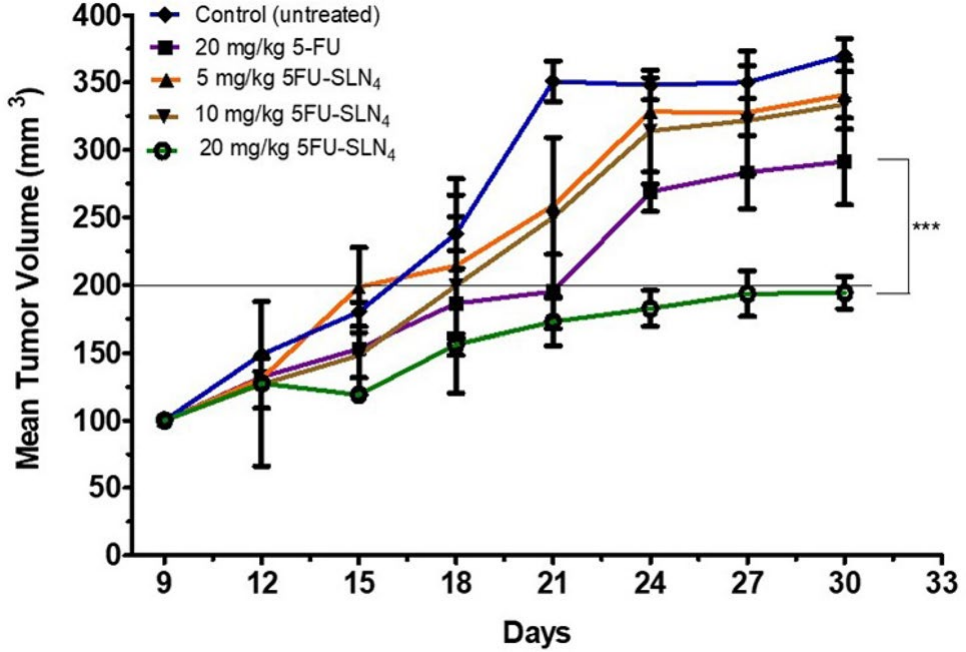
Efficacy study: Comparison of anticancer effects of 5-FU and 5FU-SLN_4_ on tumor growth of HCT-116 tumor bearing mice. Data represented as mean ± SD, (number of mice in each treatment group (n) = 4).

The tumor-inhibition study was conducted over a period of 30 days and was concluded when the normalized mean tumor volume of control (untreated) animals reached 375 ± 12 mm^3^. From Fig. 5, it was clear that 20 mg/kg 5FU-SLN_4_ was more effective in controlling the tumor growth compared to the other treated groups such as 20 mg/kg 5-FU, 5 mg/kg 5FU-SLN_4_ and 10 mg/kg 5FU-SLN_4_. However, the normalized mean tumor volumes of 20 mg/kg 5FU (300 ± 50 mm^3^), 5 mg/kg 5FU-SLN_4_ (328 ± 23 mm^3^) and 10 mg/kg 5FU-SLN_4_ (325 ± 21 mm^3^) treated groups were significantly less than the control (untreated or normal saline) tumor (375 ± 12 mm^3^). Although 5 mg/kg 5FU-SLN_4_ and 10 mg/kg 5FU-SLN_4_ treatment groups were included in the tumor efficacy study, our final goal was to investigate the efficacy of 20 mg/kg 5-FU and 20 mg/kg 5FU-SLN_4_ in controlling tumor growth. At the end of the study (30 days), mean tumor volume (200 ± 8 mm^3^) of 20 mg/kg 5FU-SLN_4_ treated group was significantly lower than the mean tumor volume (300 ± 50 mm^3^) of 20 mg/kg 5-FU treated group (*p* < 0.0001).

Recently, there have been several reports focused on the tumor doubling time (TDT) of cancer^30–32^ which sought to establish relationship between TDT and cancer prognosis which states that the longer TDT the better the prognosis. In this study, mean tumor volume of 20 mg/kg 5FU-SLN_4_ treated group took 30 days to double while mean tumor volume of 20 mg/kg 5-FU treated group doubled within 21 days. This suggests that 20 mg/kg 5FU-SLN_4_ might be more effective in suppressing tumor growth compared with 20 mg/kg 5FU. This was expected, given the short half-life of 5-FU (Table 2) which is most likely due to rapid metabolism and the fact that it was administered without any protection. While SLN_4_ acted as an efficient vehicle or delivery system, it may have afforded 5-FU excellent protection, leading to prolong systemic circulation which may lead to more 5FU-SLN_4_ to accumulate in tumor site. However, biodistribution studies need to be performed to conclusively determine whether 5FU-SLN_4_ indeed accumulate to a greater extent in the tumors relative to other tissues. There were no significant weight changes in animals among all the treatment groups during study period, indicating minimum or total lack of apparent toxicity of the administered formulations (Supplementary Fig. S4).

The in vivo evaluations in this study employed a subcutaneous xenograft versus an orthotopic model employed in the study by Tseng et al.^33^. For future investigations in orthotopic models, the surface of SLN_4_ delivery system will be grafted with ligand specific to receptors expressed on CRC cells.

### Immunohistochemistry (IHC)

**Figure 6:**
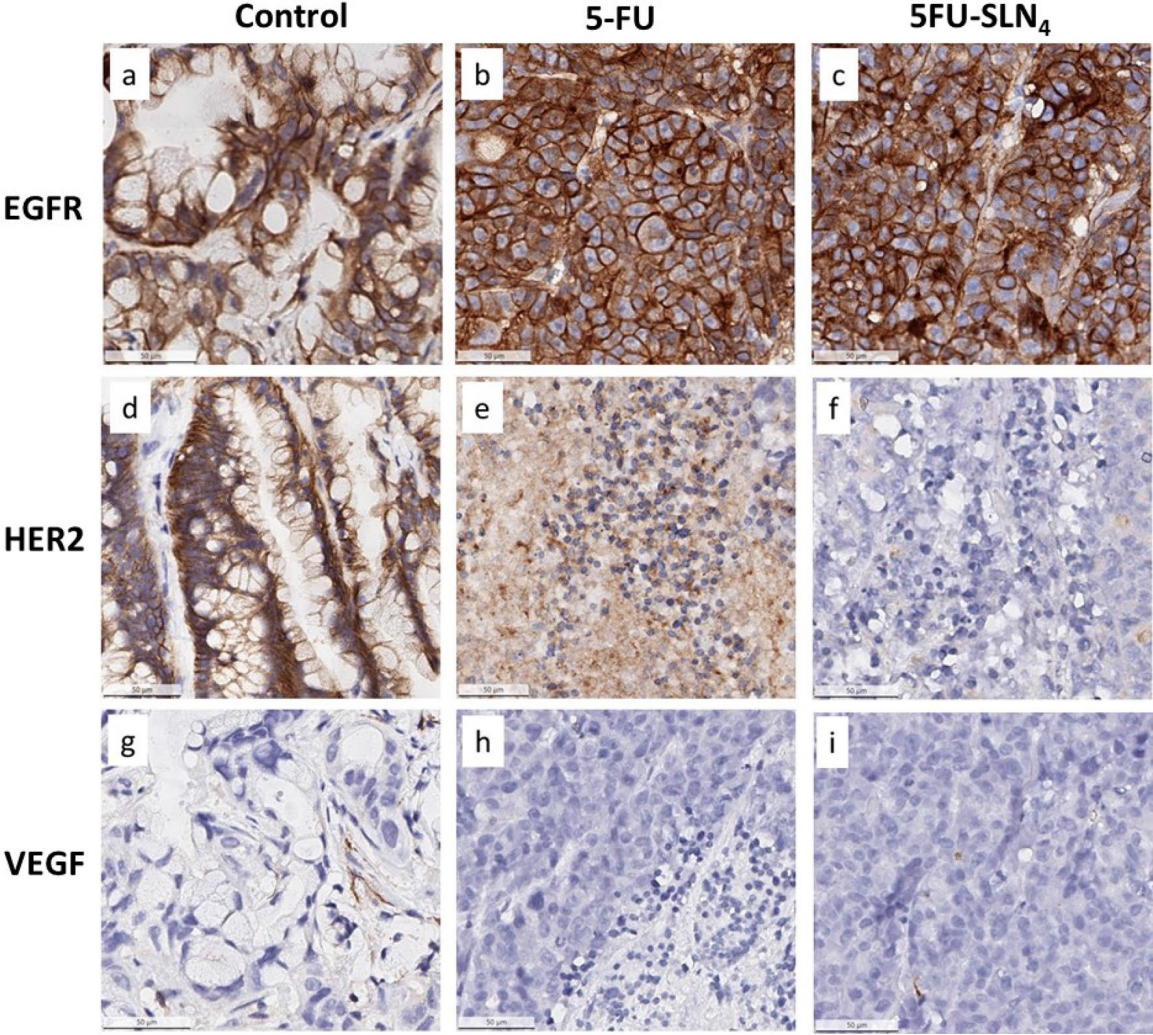
Expression of EGFR, HER2 and VEGF in colorectal cancer tissues. The protein expression of EGFR was positive (**a**), positive (**b**), positive (**c**); protein expression of HER2 was positive (**d**) positive (**e**), weak positive (**f**), and protein expression of VEGF negative (**g**), negative (**h**), negative (**i**), respectively.

Epidermal growth factor receptor (EGFR) and human EGFR 2 (HER2) and vascular endothelial growth factor receptor (VEGFR) are tyrosine kinase receptors highly expressed in several solid tumors including CRC^34–36^. These receptors are frequently reported to harbor aberrant activities that lead to the proliferation, survival, migration and differentiation required for CRC pathogenesis. We therefore evaluated the impact of our novel formulated 5FU-SLN_4_ on the expression of EGFR, HER2 and VEGFR in the mice xenograft tumors derived from HCT-116 cells (Fig. 6). Immunohistochemistry staining of the tumor tissues indicated intense expression of EGFR in the control untreated (Fig. 6a) as well as 5-FU and 5FU-SLN_4_ treated tissues (Fig. 6b,c). Based on our previous observation on the inhibitory effect of the compounds on EGFR signaling from the western blotting, the only plausible explanation to this increased EGFR expression is a possible compensatory protective response to overcome inhibition of downstream signaling in order to overcome growth suppression and to increase survival^37^.

The control untreated tissue also showed intense HER2 immunoreactivity indicating relatively high expression of the protein (Fig. 6d). Treatment with 5-FU and 5FU-SLN_4_ caused a significant decrease in HER2 expression as shown in Fig. 6. Specifically, 5-FU-SLN_4_ was more effective at reducing HER2 expression compared to 5-FU as shown by relatively low immunoreactivity to HER2 in 5FU-SLN_4_ treated tumor tissues (Fig. 6e,f). Although in general, only trace to weak immunostaining was observed in the tumor tissue for VEGFR (Fig. 6g), immunoreactivity was markedly reduced in both 5-FU and 5FU-SLN_4_ treated tumors (Fig. 6h,i). Significant differences in both HER2 and VEGFR immunoreactivity intensities between the control untreated tissues (Fig. 6d,g) and 5-FU and 5-FU-SLN_4_ treated tumor (*p* < 0.0001 and *p* < 0.001 respectively, Table 3) were observed when the stained sections were further analyzed as shown in Table 3.

**Table 3:** Differential expression of receptors in mice bearing HT-116 subcutaneous cancer after treatment with 5-FU and 5FU-SLN_4_ and staining scores analysis.

	Control	5-FU	5FU-SLN_4_	*p*-value (5-FU versus 5FU-SLN4)	Significant?
**EGFR**					
% Positive cells	76.1 ± 2.2	69.1 ± 4.1	71.4 ± 2.8	0.467	No
% Weak positive cells	45.1 ± 3.2	46.9 ± 2.5	33.4 ± 2.6		
% Moderate positive cells	24.9 ± 4.2	18.9 ± 0.2	27.3 ± 1.8		
% Strong positive cells	6.1 ± 0.9	3.3 ± 0.5	10.7 ± 0.5		
% Negative cells	23.9 ± 4.1	30.9 ± 1.7	28.6 ± 4.7		
H score	113.4	94.6	119.8		
**HER2**					
% Positive cells	80.5 ± 2.8	26.9 ± 1.4	3.7 ± 0.5	0.0001	Yes
% Weak positive cells	0.001	26.8 ± 3.6	3.6 ± 0.2		
% Moderate positive cells	10.1 ± 2.2	0.1 ± 0.002	0.02 ± 0.0		
% Strong positive cells	70.4 ± 2.8	0.006 ± 0.0	0.003 ± 0.0		
% Negative cells	20.4 ± 1.8	73.1 ± 8.6	96.3 ± 1.7		
H score	57.9	27.1	3.7		
**VEGF**					
% Positive cells	20.5 ± 4.2	1.27 ± 0.05	2.8 ± 0.4	0.001	Yes
% Weak positive cells	11.7 ± 3.2	1.23 ± 0.01	1.8 ± 0.02		
% Moderate positive cells	7.1 ± 1.6	0.04 ± 0.0	0.7 ± 0.04		
% Strong positive cells	1.8 ± 0.2	0.0003 ± 0	0.25 ± 0.001		
% Negative cells	79.5 ± 5.1	99.1 ± 1.4	97.2 ± 3.6		
H score	31.3	0.91	4.1		

## Conclusion

The development of novel delivery system capable of delivering high payload of chemotherapeutic agents across the tumor microenvironment to target cancer cells and enhance therapeutic response has been a challenge or daunting task. 5-FU has shown efficacy in the treatment of cancers but suffers from physico-chemical limitations that restrict its use as a free 5-FU; however, SLN_4_ developed in this project appeared to be robust and versatile, and seemed to have the desired requisite of novel drug delivery that could be utilized to overcome the limitations encountered by 5-FU and other free anticancer agents. Most importantly, 5FU-SLN_4_ activity in mouse bearing HCT-116 subcutaneous tumor significantly inhibited the HER2 expression. In literature, HER2 overexpression or amplification has been implicated as one of the drivers in CRC progression^34,35^. But further investigation of 5FU-SLN_4_’s biodistribution in vivo needs to be carried out to fully determine its pattern of distribution in tissues relative to tumor tissue.

Finally, the 5FU-SLN_4_ developed in this project revealed an enhanced in vitro activity, which translated into to a favorable therapeutic efficacy in vivo compared to the free 5-FU.

## Materials and methods

### Materials

Compritol 888 ATO (glyceryl behenate) and Precirol ATO 5 (glyceryl palmitostearate) were obtained from Gattefosse (Saint Priest, France). Tween 80 (polysorbate 80), Lecithin, glycerol monostearate and 5-FU were purchased from Sigma-Aldrich (St. Louis, Missouri, USA). Trevigen HT PARP/Apoptosis Assay kit was purchased from Sigma-Aldrich (St. Louis, Missouri, USA). Colorectal carcinoma cell lines, HCT -116, were obtained from American Type Culture Collection (ATCC). All other chemicals used were of an analytical reagent grade.

### Preparation of PEGylated 5-FU SLN suspension

Different 5FU-SLNs were prepared by means of cold emulsification method using high-shear mixer based on a reported method with slight modifications^38^ as illustrated in Fig. 1. A combination of PEGylated lipids and surfactants were used in the optimization of 5FU-SLN. The following lipids formed the matrix: glycerol monostearate, mPEG2000-DSPE, Compritol or Precirol (2–3% w/v ratio) and the formulations were stabilized with Tween 80, Lecithin and Poloxamer (1.5–4% w/v ratio).

Lipids with different amounts and 5-FU (0.15% w/v) in a glass vial were heated to 70–80 °C to melt. For the cold homogenization technique, the 5FU-containing lipid melt is cooled, the solid lipid ground to lipid microparticles (approximately 50–100 μm) and these lipid microparticles were dispersed in a cold surfactant solution (water, Tween 80, Lecithin or Poloxamer) to yield a pre-suspension. This pre-suspension was homogenized (1,200 rpm (3,820 rcf), 3–5 cycles) on ice-bath (below room temperature) causing lipid microparticles to break directly to solid lipid nanoparticles. This process avoids or minimizes the melting of the lipid and therefore minimizing loss of hydrophilic drugs to the water phase^39^. The freshly prepared formulations were used to characterize all the various SLN formulations and, for in vitro and in vivo studies. The selected SLN formulation (5FU-SLN_4_) was lyophilized with 5% w/v mannitol and stored at 4 °C for future use.

### Characterization of SLN formulations

#### Transmission electron microscopy (TEM)

The morphological examination of the PEGylated 5FU-SLN_4_ was performed using high resolution TEM. The 3 μl samples were stained with 50 μl of ammonium molybdate solution (1% w/v) after adjusting the 5FU-SLN_4_ suspension to pH of 7.0 with 5 N sodium hydroxide. The stained sample was then placed on copper grids, allowed to dry and viewed by Tecnai F-20 transmission electron microscope (Philips Co. Japan).

#### Particle size and zeta potential

All analyzed samples were diluted with deionized water for both particle size and zeta potential measurements using a Zeta Potential/Particle Sizer (NICOMP 380 ZLS). All measurements were performed in triplicate. The NICOMP 380 ZLS measures the nanoparticles based on principles of dynamic light scattering (DLS).

#### HPLC analysis

5-FU analysis was performed according to method described by Ciccolini and colleagues with minor modification^40^. Briefly, 5-FU analysis was performed using a chromatographic system, which consisted of a HPLC (Waters Corporation, Milford, MA) equipped with an auto-sampler, photo diode array (2998 UV/Vis) detector and pumps. Separation was performed using a reverse phase column (ZORBEX SB—C18 4.6 × 250 mm, 5 μm).

A flow rate of 1.0 mL/min and injection volume 20 μl at ambient temperature were maintained while detection was performed at wavelength of 268 nm^41^. Prior to analysis, reverse phase column was equilibrated with mobile phase made up of methanol 5 mM sodium phosphate buffer in ratio of 595, and pH adjusted to 5^42^. An isocratic elution was performed throughout the entire analysis including internal standards.

A calibration curve was prepared using 5-FU standard solutions with concentration range of 3–100 µg/mL (r = 0.9). A plot of the peak areas as a function of 5-FU concentration was plotted and the linear equation of the calibration curve given as y = mx + c was determined, where y is the peak area, m is the slope, x is the concentration of 5-FU and c is the y—intercept. Supernatants from controls were spiked with aliquots of 0.5 μg/ml of 5-FU.

### Encapsulation efficiency (%)

The entrapment efficiency (E.E) corresponds to the amount of drug incorporated in the nanoparticle. Entrapment efficiency analysis was performed by adding 1 mL of loaded SLN to 1 mL of methanol. The mixture was placed into the chamber of the ultrafiltration tube and centrifuged at 6000 rpm for 6 min. After centrifugation, the non-entrapped drug amount in the supernatant was collected and ran on HPLC analysis to estimate the amount of 5-FU present in the sample. The E.E was calculated using the following equation:

1 $$E.E = \frac{{{\text{Total amount of drug}} - {\text{Free drug}}}}{{\text{Toal amount of drug}}} \times 100$$

### In vitro release of 5-FU

The cumulative release of 5-FU from the solid lipid nanoparticles was performed at 37 ± 1 °C. Twenty milligrams (20 mg) of lyophilized 5FU-SLN_4_ was suspended in 1.0 ml of PBS (pH = 7.4), transferred into a dialysis bag (MWCO = 3,500) and incubated in 5 ml of PBS pre-equilibrated to temperature (37 ± 1 °C). One milliliter (1.0 ml) was collected at selected time points and replaced with 1.0 ml of fresh PBS. Collected samples were analyzed by HPLC–UV/Vis spectrophotometry to determine the amount of 5-FU released at different time points^43^.

### Mathematical models to determine 5-FU release kinetics

The release kinetics of 5-FU from the heat sensitive liposomes was investigated to predict the possible mechanism of release using mathematical models. The release order was determined using zero order (Eq. 2) and first order kinetic model as shown below.

2 $$C={C}_{o}+{K}_{o}t$$

3 $$Log C=Log {C}_{o}+\frac{{K}_{1}t}{2.303}$$

where C_o_ is the initial amount of drug, C is the % cumulative 5-FU released (zero order) or first order (Eq. 3) at time “t” and K_o_ is zero order release constant and K_1_ is the first order release constant^44^.

A Higuchi model was used determine whether the release mechanism follows Fickian diffusion as shown below^20^:

4 $$C= {C}_{o}+{K}_{H}{t}^{1/2}$$

where C is the % cumulative 5-FU release at time, t and K_H_ is the Higuchi constant.

### Cytotoxicity of PEGylated 5FU-SLNs against HCT-116 cells

*Cell Viability*: Cell viability study was performed using 96-well plates and evaluated by Alamar Blue assay. The HCT-116 cells were seeded at a density of 1 × 10^3 ^cells/well. The cells were cultured in Dulbecco's Modified Eagle Medium (DMEM)/F-12 medium enriched with L-glutamine, HEPES, 10% fetal bovine serum (FBS), and 1% penicillin streptomycin. Cells were maintained at 37 °C and 5% CO_2_. After cells were grown to 70% confluence, they were treated with freshly prepared treatments at different concentrations of 5-FU-SLN and 5-FU equivalents. Cells were treated for a 48-h period followed by termination, stained with 0.05% resazurin, gently mixed and incubated in the dark for 2–3 h before measurement of the fluorescence with excitation at 560 nm and emission at 590 nm using GloMax Explorer Microplate Reader (Promega Biotech, Madison, WI).

### Cellular uptake of FITC-labeled PEGylated SLNs

#### Confocal imaging

HCT-116 cancer cells were grown in 6-well plates (with cover slips) at a cell density of 2 × 10^3^, for 24 h at 37 °C^45^. The cells were then treated with fluorescein isothiocyanate (FITC)-labeled SLN_4_ in growth media. After 3 h, FITC-SLN_4_ was removed and the cells gently washed twice with PBS. Next, 5 μg/ml of Hoechst dye was added for nuclear staining, the cells were fixed using 4% paraformaldehyde, then mounted and imaged using Leica SP2 Multiphoton system^45^.

#### Flow cytometry

To determine 5-FU-loaded solid lipid nanoparticle uptake by the cells, HCT-116 cells were grown in 6-well plates at a cell density of 1 × 10^5^ and cultured in growth media until 70% confluence^45^. Cells were treated with the rhodamine-labeled solid lipid nanoparticles (Rho-SLN) for 24 h at 37 °C. After treatment, the adherent cells were washed three times with PBS, detached from the culture plate with 0.25% trypsin- ethylene diamine tetraacetic acid (EDTA) solution, and centrifuged at 6,000 rpm (3820 rcf) for 5 min^45^. Finally, the cells were re-suspended in 500 μl PBS, fixed with 4% paraformaldehyde, and kept on ice until analysis using a Becton Dickinson (BD) Fluorescence-Activated Cell Sorting Canto Analyzer and a BD Fluorescence-Activated Cell Sorting Aria Cell Sorter (BD Biosciences)^45^.

### Clonogenic study

For colony assay, HCT-116 cells were seeded into T-25cm^2^ culture flask at a density of 5 × 10^5^ cells and cultured in DMEM/F12 medium supplemented with 2 mM L-glutamine, 10 mM HEPES, 10% FBS, and 1% penicillin/streptomycin^45^. After the cells reached 70% confluency, they were exposed to different concentrations of free 5-FU and 5FU-SLN_4_^45^. Treatment was repeated after 24 h for 48 h exposure after which the experiment was terminated, cells harvested, and then re-plated onto 6-well plates at a density of 1,000 cells per well and incubated with growth medium for 7 days^45^. After the control cells reached 75% confluence, the experiment was terminated by fixing and staining the plates with 0.5% crystal violet solution^45^. The stained colonies (fifty per colony) were counted using a Jenco Stereomicroscope.

### Western blot analysis

Western blot analysis were conducted by seeding (1 × 10^6^) HCT-116 cells into 75 cm^2^ flask. The cells were treated with 3, 6 and 12 µM of 5FU-SLN_4_. After a 48 h period, treatment was terminated through trypsinization, cell pellets were collected after centrifugation and washed 3 times. The cell pellet was collected in microcentrifuge tubes and lysed with RIPA buffer supplemented with 1% Protease inhibitor cocktail (Millipore Sigma, St Louis, MO). Samples were centrifuged for 20 min at 14,000 rpm (3,820 rcf) and the supernatant stored at − 20 °C until further used. Lysates containing 100 μg of protein were mixed with SDS-PAGE sample buffer, placed in boiling water bath for 5 min and separated by Sodium dodecyl sulfate polyacrylamide Gel Electrophoresis (SDS-PAGE) and proteins were transferred onto nitrocellulose membranes (Bio-Rad, CA) overnight^46^. After blocking, membranes were incubated with primary antibodies at 4 °C overnight and then horseradish-peroxidase (HRP)-conjugated anti-mouse secondary antibodies (1:1,000; Cell Signaling, MA, USA) were added for 1 h at RT^46^. Chemiluminescence images were captured using the ChemiDoc Imaging System (Bio-Rad, CA).

#### Cutting of membranes

To enhance the detection of multiple proteins, membranes were cut into individual strips for better detection with different antibodies prior to imaging.

Western Blot bands were quantified by densitometric analysis using Image Lab software (Bio-Rad Laboratories) and GraphPad Prism version 6. The primary and secondary antibodies were: EGFR (1:1,000, sc-373746; Santa Cruz Dallas, TX), pEGFR (1:1,000, 2234) (Cell Signaling, MA), STAT3 (1:1,000, 9139; Cell Signaling, MA), pSTAT3 (1:1,000, 9134; Cell Signaling, MA), AKT (1:1,000, 9272; Cell Signaling, MA), pAKT (1:1,000, 9271; Cell Signaling, MA). Anti-Beta-Actin (1:1,000, sc-517582; Santa Cruz Dallas, TX) served as loading control for protein normalization. The experiments were performed at least two times.

### Animal studies

NOD/Scid mice were obtained from Jackson Laboratory (Bar Harbor, ME) and housed in a virus-free, indoor, light and temperature-controlled environment provided with adequate food and water. All procedures with animals were in accordance with the National Institutes of Health Guide for the Care and Use of Laboratory Animals and approved by the Florida A&M University Animal Care and Use Committee. Colorectal HCT-116 cancer cells were cultured in DMEM/F12 medium supplemented with 10% FBS and 1% penicillin-streptomycin and incubated in 5% CO_2_ at 37 °C^47^.

Mice were inoculated with HCT-116 cells (5.7 × 10^6^ in 150 μl PBS) and injected subcutaneously in the lower flanks of NOD/Scid mice^48,49^. Prior to the tumor appearance within two weeks of inoculation, mice were grouped into 6 groups with 5 mice in a group [control, 5-FU, 5FU-SLN_4_ (5 mg/kg), 5FU-SLN_4_ (10 mg/kg) and 5FU-SLN_4_ (20 mg/kg)]. The mice were administered with selected formulations for 2 weeks, every other day via intraperitoneal (i.p.) injection^50^. The control group received normal saline throughout the study. Tumor measurements were taken every other day using a digital Vernier caliper throughout the experiment. Mice were euthanized when tumor size reached 2.0 cm.

#### Pharmacokinetic studies

A group of mice were injected intraperitoneally with a single dose of 20 mg/kg of 5-FU and 5FU-SLN_4_ (at 5-FU equivalent dose). Blood samples were taken from the tail vein of mice at different time points (5 min, 10 min, 15 min, 1, 2, 4, 8, 12 and 24 h). The samples were placed in heparinized centrifuge tubes and centrifuged at 12,000 rpm for 8–10 min. The plasma was then stored at − 20 °C until analysis.

#### Assay validation and recovery

A calibration curve was prepared using the peak areas of 5-FU against the respective concentrations of 5-FU. Linear regression analysis of the calibration data was performed using the equation y = mx + c, where y is the peak area, x is the concentration of 5-FU, and m and c are the slope and intercept, respectively^51^. The recovery of 5-FU from plasma or tissue samples was determined by comparing the peak areas from the samples containing known added 5-FU with those from 5-FU standard solutions^51^.

### Immunohistochemistry

Mice were euthanized and tissue samples (tumor, liver and kidney) were excised from mice immediately, washed with PBS and placed in 10% buffered formalin for 24 h and transferred to 70% ethanol for histopathological analysis. Histology was performed by HistoWiz Inc. (histowiz.com) using a Standard Operating Procedure and fully automated workflow. Samples were processed, embedded in paraffin, and sectioned at 4 μm^52–54^. Immunohistochemistry was performed on a Bond Rx autostainer (Leica Biosystems) with enzyme treatment (1:1,000) using standard protocols. Antibodies used were rat monoclonal F4/80 primary antibody (eBioscience, 14-4801, and 1:200) and rabbit anti-rat secondary (Vector, 1:100)^52–54^. Bond Polymer Refine Detection (Leica Biosystems) was used according to manufacturer’s protocol. After staining, sections were dehydrated and film coverslipped using a Tissue-Tek Prisma and Coverslipper (Sakura). Whole slide scanning (40 ×) was performed on an Aperio AT2 (Leica Biosystems).

### Statistical analysis

Pharmacokinetic parameters were estimated using software Pharmacokinetic Solutions 2.0 (PK Solver). The difference between 5-FU and 5FU-SLN_4_ treatment groups were analyzed using paired Student’s t-test and considered significant at *p* < 0.05. All experiments were performed at least in triplicate and analyzed using GraphPad Prism software (GraphPad Software, Inc., La Jolla, CA, USA).

## Supplementary information

Supplementary Information.

## Data Availability

All data generated or analyzed during this study are included in this published article and its Supplementary Information Files.
