# Supplementary material for: Application of smart solid lipid nanoparticles to enhance the efficacy of 5-fluorouracil in the treatment of colorectal cancer
Source: Sci Rep. 2020 Oct 12;10:16989. doi: 10.1038/s41598-020-73218-6 (PMC7552424; doi:10.1038/s41598-020-73218-6)
Supplement: Supplementary file 1 — Supplementary Information. [file 41598_2020_73218_MOESM1_ESM.docx]

Supplementary information

**Application of smart solid lipid nanoparticles to enhance efficacy of 5-Fluorouracil in the treatment of colorectal cancer.**

Authors:

Taylor Smith^1^, Kevin Affram^1^, Ebony L. Nottingham^1^, Bo Han^2^_,_ Felix Amissah^3^, Sunil Krishnan^4^, Jose Trevino^5^, Edward Agyare^1^*

Affiliations:

^1^ College of Pharmacy and Pharmaceutical Sciences, Florida A & M University, Tallahassee, Florida, USA

^2^ Department of Surgery, Keck School of Medicine University of Southern California, Los Angeles, California, USA

^3^ College of Pharmacy, Ferris State University, Big Rapids, Michigan, USA

^4^ Mayo Clinic, Jacksonville, Florida, USA

^5^ Department of Surgery, College of Medicine, University of Florida, Gainesville, Florida, USA

**Supplementary Figure S1**

**Supplementary Figure S2**


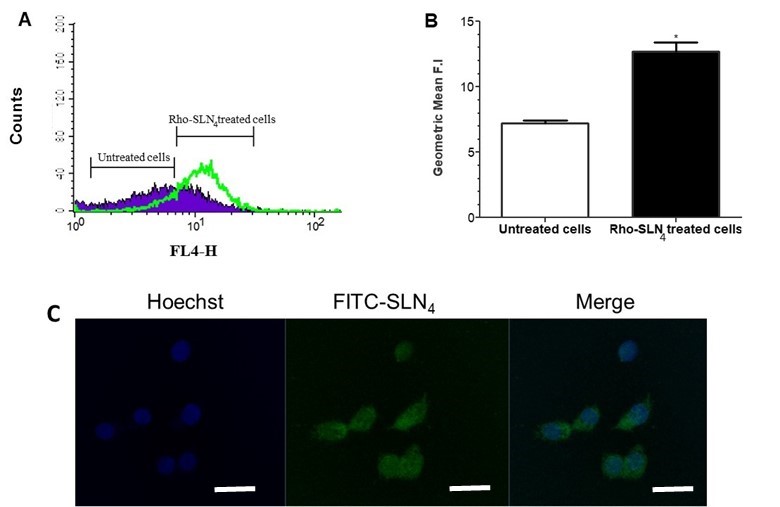


**Supplementary Figure S3**


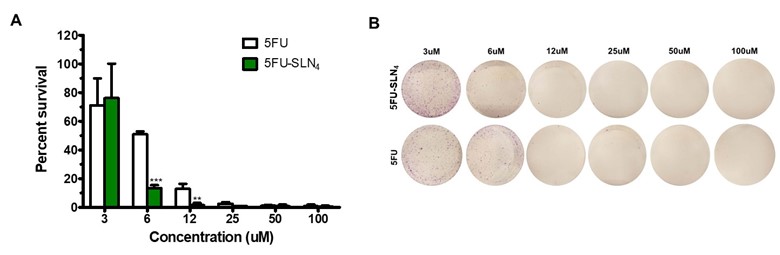


**Supplementary Figure S4**

**
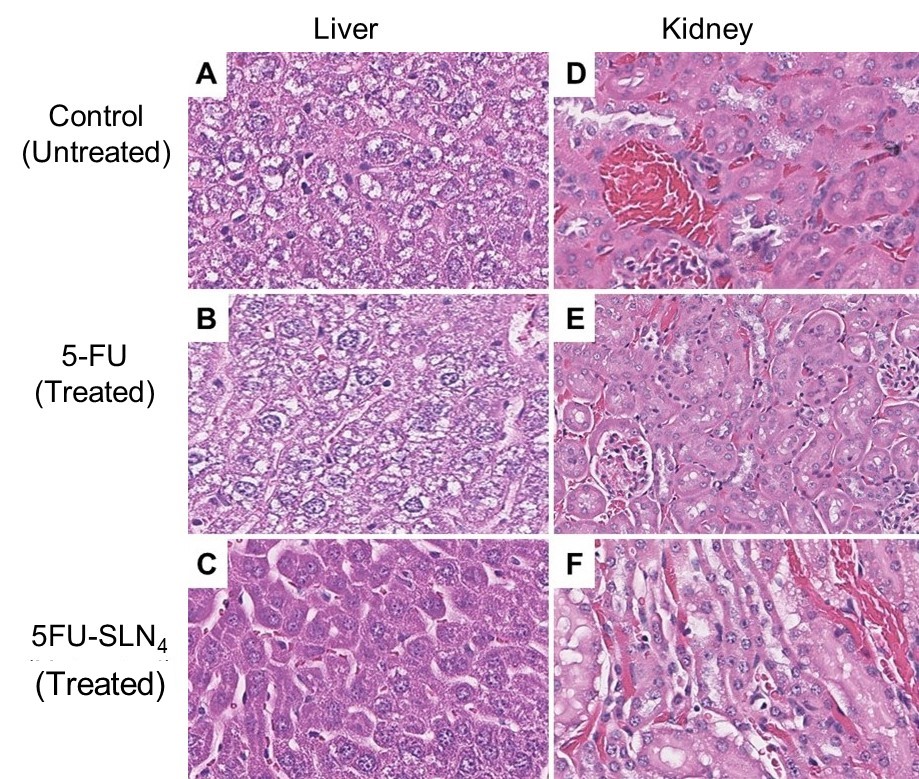
**

**Supplementary Figure S5**


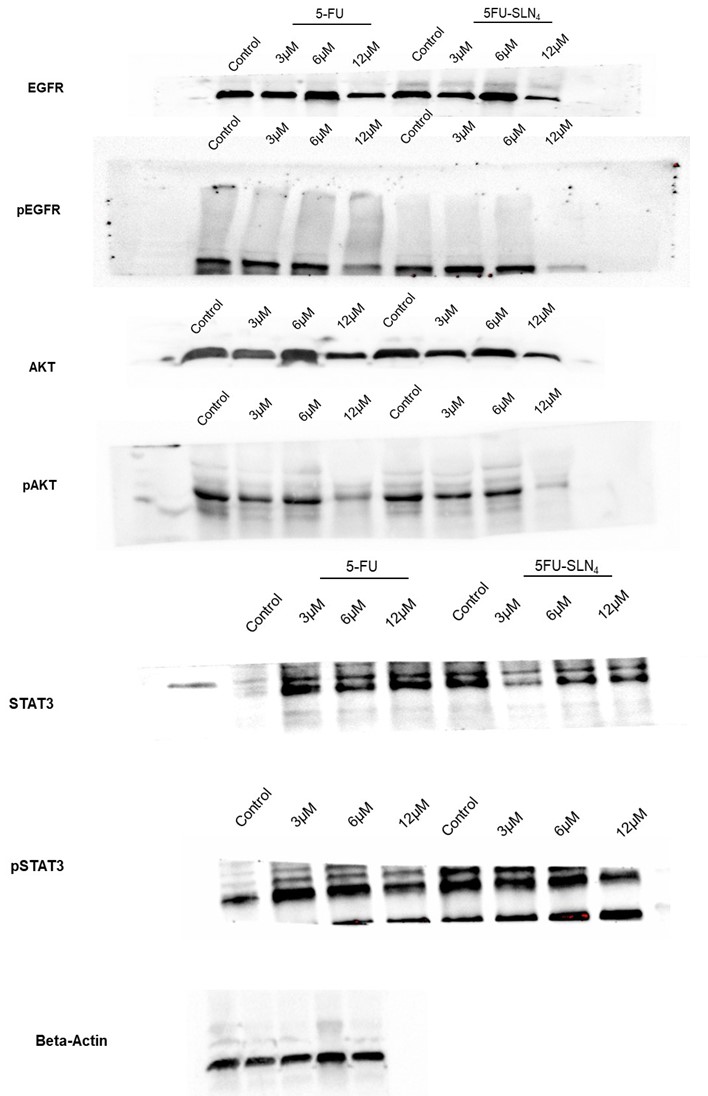


**Supplementary Figure S6**

**Supplementary Figure S7**

**
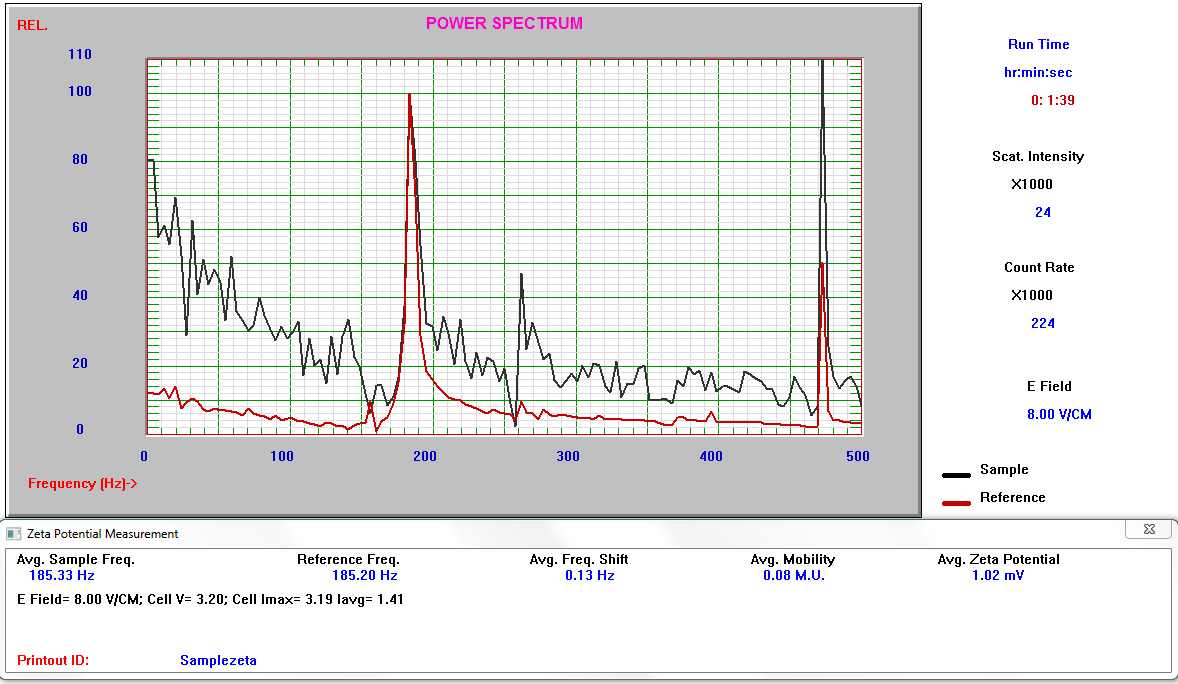
**

**Supplementary Figure S8**

**
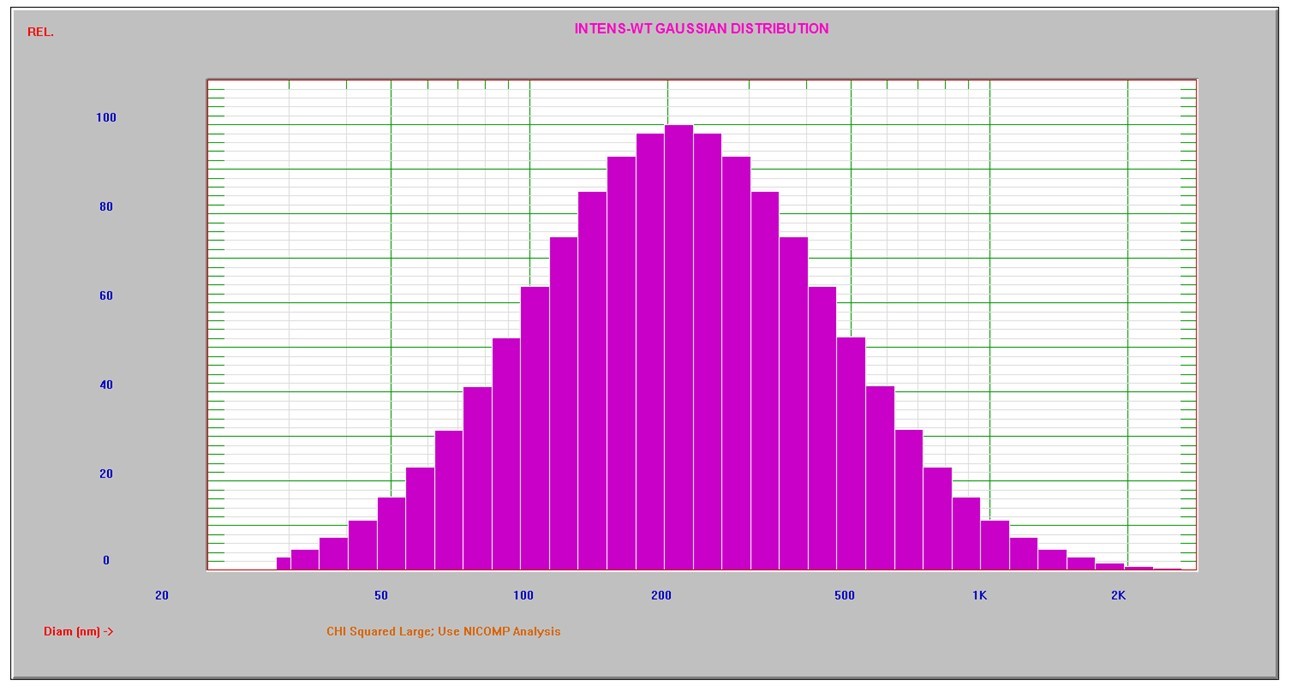
**

**Supplementary Figure Legends**

**Supplementary Figure S1**

Transmission electron micrograph of 5FU-SLN_4_ (Scale bar = 400 nm)

**Supplementary Figure S2**

**Cellular uptake studies:** (A) Flow cytometry analysis of Rho-SLN_4_ on HCT-116 cells, (B) Geometric mean of fluorescence intensity comparing cellular uptake of Rho (untreated HCT-116 cells) and Rho-SLN_4_ against HCT-116 cells (*p<0.05), and (C) Confocal microscopy images showing cellular uptake of FITC-SLN_4_ by HCT-116 cells after incubation for 3 h. The nucleus was stained by using the DNA-binding dye Hoechst 33342 (shown in blue) after cell fixation and the presence of FITC-SLN_4_ in cells labeled in green. Merged image exhibits the co-localization of FITC-SLN_4_ and nucleus in HCT-116 cell. (Scale bar =10 µm)

**Supplementary Figure S3**

**Clonogenic assay for 5-FU and 5FU-SLN_4_ treated HCT-116 cells:** (A) Survival percent, and (B) Colony images after cells were treated with 5-FU and 5FU-SLN_4_ at 3-100 µM. For statistical comparison between 5-FU and 5FU-SLN_4_ treated groups, p-value was calculated by student’s t-test, (5-FU vs 5FU-SLN_4_, **p<0.01 and ***p<0.001).

**Supplementary Figure S4**

**H&E images:** Liver from untreated mouse (control) (A), Liver from mouse treated with 5-FU dose of 20 mg/kg (B), Liver from mouse treated with 5FU-SLN_4_  (dose equivalent 5-FU, 20 mg/kg) (C), Kidney from untreated mouse (D), Kidney from mouse treated with 5-FU (20 mg/kg) E), Kidney from mouse treated with 5FU-SLN_4_ (dose equivalent 5-FU, 20 mg/kg). (Magnification X 40)

**Supplementary Figure S5.** Uncropped full-length western blot images. Shown are the original blots obtained prior to cropping for better visualization. Membranes were often cut to enable blotting for multiple antibodies. Beta-actin level was used as a loading control for the Western blot to normalize the levels of protein detected by confirming that protein loading is the same across the gel.

**Supplementary Figure S6.** Cell Viability of HCT-116: Cell viability after exposure of the HCT-116 cells to SLN_4_ (Blank) and varying concentration of 5-FU and 5FU-SLN_4_ for 72 h (** p<0.01, *** p<0.001 (Student's t-test))

**Supplementary Figure S7.** Graph of zeta potential distribution for 5FU-SLN_4_

**Supplementary Figure S8.** Dynamic light scattering (DLS) graph showing hydrodynamic particle size distribution of freshly prepared 5FU-SLN_4_ with mean diameter of 263 nm.

| **Formulation** | **Lipid** | **Lipid Ratio**  **(%w/v)** | **Surfactants Composition** | **Surfactant Ratio**  **(%w/v)** | **Mean Particle Size (nm)** | **Zeta Potential (mV)** | **EE (%)** |
| --- | --- | --- | --- | --- | --- | --- | --- |
| SLN_4_ | Precirol | 3 | Tween80/Lecithin | 4:2 | 173 ± 9 | 0.8 ± 0.01 | BLK |
| 5FU-SLN_4_ | Precirol | 3 | Tween80/Lecithin | 4:2 | 263 ± 3 | 1.1 ± 0.02 | 81±10 |

**Supplementary Table S1.** Comparison of blank SLN and the desired 5FU-SLN_4_ formulation

BLK = Blank or empty SLN_4_
